# A Series of Four Cases of Swallowing Artificial Teeth Treated in the Royal Southern Hospital, Liverpool, during the Last Six Months

**Published:** 1898-10

**Authors:** John Owen


### A SERIES OF FOUR CASES OF SWALLOWING ARTIFICIAL TEETH TREATED IN THE ROYAL SOUTHERN HOSPITAL, LIVERPOOL, DURING THE LAST SIX MONTHS.

BY JOHN OWEN, M.B.

THE following series of cases of swallowing artificial teeth may prove of interest:

CASE I.—William P., aged thirty, admitted to hospital at noon, March 20, 1898, complaining of having swallowed his false teeth during sleep twelve hours previously. He was suffering from dysphagia, aphonia, and slight dyspnœa.

On examination the pomum Adami was found slightly displaced to the right, and there was a fulness to the left of the larynx about the level of the cricoid. No foreign body could be felt by the introduction of the finger into the pharynx; œsophageal bougies could not be passed beyond the level of the larynx.

Extraction with forceps being deemed inadvisable, œsophagotomy was decided upon. The operation was easily and successfully carried out and the teeth extracted from their position at junction of pharynx and œsophagus without any difficulty. The edges of the œsophageal opening were brought together by two silk sutures. The wound was cleansed and the skin sutured, an opening being left for drainage at the most dependent part.

In spite of the most careful after-treatment the wound went wrong on the third day, and he eventually succumbed to septicæmia twelve days after the operation. The plate was unfortunately lost. It was made of metal and carried four teeth. The hooks were unbroken when the plate was extracted.

CASE II.—Charles H., aged fifty-six, admitted into hospital May 6, 1898, with similar history to above, but was unable to reach hospital until thirty-six hours after the accident. When first seen he was suffering from marked dysphagia and aphonia and some slight dyspnœa. He was unable to swallow fluids and could not speak above a whisper.

Being a stout, thick-necked man there was no external appearances to indicate the position of the foreign body; it could, however, be felt by the tip of the finger down the pharynx.

An X-ray examination was made by Dr. Holland, the plate being seen on the screen. With considerable difficulty the plate was extracted by means of laryngeal forceps. The after-treatment consisted in keeping the bruised parts at rest and application of warm fomentations to the neck. His condition steadily improved during the subsequent forty-eight hours, the breathing becoming easier, with an entire absence of any signs of œdema of the glottis.

At this stage he for the first time complained of a sinking feeling, became restless, sat up in bed, and fell back dead in twenty minutes after the onset of this feeling of faintness. There was no post-mortem examination, but death was evidently due to syncope.

The man's appearance led to the assumption that fatty degeneration of the heart had a great deal to do with the fatal attack of syncope. The plate was made of metal, and carried six teeth.

Case III.—James W., aged nineteen, admitted to hospital January 6, 1898, stating that he woke up in the morning with a pain in the throat and inability to swallow. He had retired the previous night wearing his false teeth, which he missed on awakening.

There was no external evidence of the situation of the plate, and no foreign body could be felt by the finger in the pharynx. A bougie was passed well beyond the level of the larynx before meeting with any resistance, and this resistance was without much difficulty suddenly overcome, the bougie now passing into the stomach. Immediately afterwards he was able to swallow. He was kept under observation for a month, during which time he showed no untoward symptom and did not pass the plate.

On making inquiries from his medical attendant, I find that the patient has since remained perfectly well and is not aware of having passed the plate per rectum. The plate was made of vulcanite and was small, carrying but two teeth.

Case IV.—Mrs. R., aged thirty-five, came to out-patient department April 20, complaining of pain behind the sternum at level of xiphoid cartilage, and of having swallowed her false teeth the previous day, owing to a fall.

No indication of their wherabouts could be got by examination through the screen with X-rays or by skiagram. An œsophageal bougie could be passed to the stomach without impinging on a foreign body. Diet of solid nature was ordered and patient was sent home. On the third day from first swallowing the plate (two days from first coming to hospital) the patient brought the plate, which had been passed per rectum the previous evening. It was made of vulcanite, with two sharp metal hooks, and carried four incisor teeth, the longest diameter being one and one-fourth inches. —*Journal of the British Dental Association.*

